# Micro-3D sculptured metastructures with deep trenches for sub-10 μm resolution

**DOI:** 10.1038/s41378-025-00888-5

**Published:** 2025-03-12

**Authors:** Anıl Çağrı Atak, Emre Ünal, Hilmi Volkan Demir

**Affiliations:** 1 Department of Electrical and Electronics Engineering, Department of Physics, UNAM – National Nanotechnology Research Center and Institute of Materials Science and Nanotechnology, Bilkent University, Ankara, 06800 Turkey; 2 Luminous! Center of Excellence for Semiconductor Lighting and Displays, School of Electrical and Electronic Engineering, Division of Physics and Applied Physics, School of Physical and Mathematical Sciences, School of Materials Science and Engineering, Nanyang Technological University, Singapore, 639798 Singapore

**Keywords:** Electrical and electronic engineering, Electronic devices

## Abstract

Three-dimensional (3D) printing allows for the construction of complex structures. However, 3D-printing vertical structures with a high aspect ratio remains a pending challenge, especially when a high lateral resolution is required. Here, to address this challenge, we propose and demonstrate micro-3D sculptured metastructures with deep trenches of 1:4 (width:height) aspect ratio for sub-10 µm resolution. Our construction relies on two-photon polymerization for a 3D-pattern with its trenches, followed by electroplating of a thick metal film and its dry etching to remove the seed layer. To test the proposed fabrication process, we built up three-dimensional RF metastructures showcasing the depth effect as the third dimension. Using the numerical solutions, we custom-tailored these metastructure resonators to fall within a specific resonance frequency range of 4-6 GHz while undertaking comparative analyses regarding overall footprint, quality factor, and resonance frequency shift as a function of their cross-sectional aspect ratio. The proposed process flow is shown to miniaturize metal footprint and tune the resonance frequency of these thick 3D-metastructures while increasing their quality factor. These experimental findings indicate that this method of producing trenches via 3D-printing provides rich opportunities to implement high-aspect-ratio, complex structures.

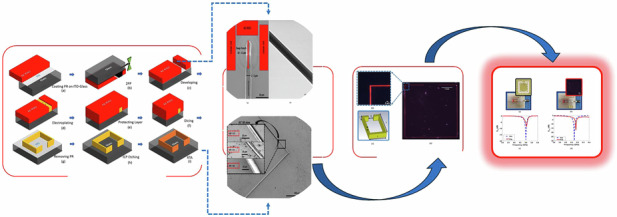

## Introduction

Three-dimensional (3D) printers, based on two-photon polymerization (2PP), offer the ability to overcome the fabrication limitations of complex structures. 2PP, a non-linear optical process that generates sufficient energy to induce a chemical reaction in the focused region of a photoactive polymer material through the simultaneous absorption of two lower-energy photons, may enable the creation of 3D surfaces with high accuracy and small feature sizes (potentially below 100 nm)^1^. The idea of two-photon absorption first emerged with Maria Goeppert-Mayer^2^. After five decades, the first 3D micro-fabricated structures were established experimentally with the stimulation of two-photon absorption^3^. Today 2PP finds use in a wide range of potential applications^4^ including those of plasmonic metamaterials^5^, helical photonic materials^6^, microfluidic channels^7^, biological lab-on-a-chip systems^8^, and micro-electromechanical systems^9^.

The versatility of 3D-printing techniques enables a diversity of application domains. Their high capability enables the adaptation of various methodologies to fulfill specific demands and technical requirements. However, having high-aspect-ratio features is still a pending challenge^10^. On the other hand, 2PP may possibly allow for the fabrication of high-aspect-ratio structures with supplemental benefits of having local polymerization with the nonlinear and ground-up process to construct intricate structures^11,12^.

In addition to these evaluations, while two-photon polymerization (2PP) has some constraints, such as low throughput and the need for advanced optimization techniques in large structures, it offers several significant advantages. It is cost-effective compared to conventional lithographic techniques, as it does not require expensive equipment like electron beam or high-vacuum systems. Furthermore, 2PP provides a broad range of material options and eliminates the need for a hard mask or mold, offering greater flexibility in fabricating complex structures. These advantages make 2PP an attractive alternative for various applications.

Other conventional lithography techniques, including electron beam lithography (EBL), nanoimprinting lithography (NIL), and optical lithography, also possess the capability to fabricate high-aspect-ratio structures, but with undesired limitations. The choice of photoresist used in these lithography techniques is critical, as the removal of negative photoresists can be challenging without the use of additional chemicals or etchants. In addition, forming high-aspect-ratio metal structures through metal deposition following the lithography process is severely limited in terms of the total thickness of the structure, working footprint, and/or patterning capability. Electron beam lithography (EBL) can be used to fabricate high aspect ratio structures. For thinner structures, such as those 600 nm, 1100 nm, and 1000 nm thick, aspect ratios of 30, 20, and 17 are achieved, respectively. Thicker structures, around 2000 nm in thickness, results in around aspect ratio of 10^13–19^. These results demonstrate that EBL has limitations in terms of total thickness and footprint. While high-aspect-ratio metal patterns are found in some of these studies, even an aspect ratio of 1:1 presents technical challenges in the micrometer range, as fabricating structures with a total thickness exceeding 2 µm is not feasible^20–22^. While the NIL technique can produce high aspect ratio structures exceeding 10 in the sub-micron range, its capability decreases as the thickness increases, yielding an aspect ratio of 4 when the total thickness is around 2 µm^23,24^. The overall thickness obtained using optical lithography is more remarkable than those of EBL and NIL^25,26^. However, their sidewalls are not perpendicular to the plane of the used substrate, caused by the proximity effect. The diffraction limit of optical lithography can cause such serious distortions in the patterning procedure.

Using our fabrication process flow of the proposed micro-sculptured deep trenches, we constructed a new class of high-aspect-ratio and sub-10 μm resolution RF metastructures that apply a third dimension to the conventional two-dimensional micro-fabricated metamaterials. As a proof-of-concept demonstration to showcase the capability of our fabrication approach, we chose metastructures in the form of split ring resonators (SRRs) as the test model and demonstrate the significance of high-aspect-ratio metal structures generally ignored, in analyzing SRRs. In the construction stage of the fabrication process flow, we designed a class of RF metastructures possessing a high-aspect-ratio up to 1:4 (width:height) at sub-10 μm resolution that makes use of the third dimension.

RF metamaterials have been used for numerous purposes including sensing^27^, material characterization^28^, and miniaturization^29^. However, their fabrication methods are limited to well-known methods including printed circuit board (PCB) writing, optical lithography, and electron beam lithography. These conventional methods suffer a number of limitations and problems with the patterning capability and resolution, limiting RF structure designs not only in the lateral dimensions. Our proposed fabrication flow is useful to overcome these issues. Furthermore, adding new capabilities to the fabrication skills provides the advantage of enhanced quality factor, miniaturization capacity, and tuning resonance frequency.

Quality factor (Q-factor) is a criterion to evaluate the frequency selective performance of RF resonators^30^. The higher Q-factor benefits the sensor technology since this typically determines sensor performance and sensitivity. Miniaturization makes the structure’s dimension smaller, which provides the cost efficiency and likelihood of MEMS design with limited footprints^31^. This means that miniaturization can provide a smaller design with the same Q-factor range and characteristic features, as in our case. In addition, it is crucial to consider the resonance frequency of a resonator, as well as the impact of its geometrical factors on its resonance frequency shift. These factors play an important role in determining the operating spectral range of the resonator. In recent years, there have been studies taking into account the effect of the finite conductive thickness of the resonator^32–34^ to tune the resonance frequency. Therefore, careful analysis and consideration of these factors with the fabrication process flow are necessary to ensure the effective operation of the resonator.

In this work, we developed our fabrication process flow based on 3D-printing along with electroplating and dry etching. Numerical solutions helped us to design proof-of-concept RF metastructures with desired dimensions. After analyzing numerical solutions, we fabricated the designed RF metastructures. In addition to enhancing design capabilities and enabling the production of high-aspect-ratio patterns, the deep-trench approach facilitated observation and analysis of changes in the Q-factor and resonance frequency. It also provided dimension miniaturization for the RF designs while considering their metal thickness and width concerning resonance frequency, footprint size, and Q-factor. Constructing different metal ratios with micro-3D sculptured deep trenches functionality enabled us to build metastructures having smaller footprints, and higher Q-factors, allowing for resonance frequency tuning as desired.

## Result and discussion

As a systematic approach to demonstrate the proposed fabrication process flow and depth effect of the RF metastructure resonators, we designed the resonators using numerical solutions within a specific resonance frequency range of 4-6 GHz. It is well known that both geometrical and material factors influence the resonance frequency of split-ring resonators. Geometrically, the gap in the split part and the side length of the resonator affect the frequency. A larger gap results in a higher resonance frequency by altering the capacitive effect, while a longer resonator decreases the frequency due to its inductive behavior. Additionally, the choice of substrate material for the resonator impacts the frequency based on its permittivity and permeability. Specifically, a substrate with higher permittivity can lower the resonance frequency. In this work, we analyzed the numerical solution results regarding miniaturization, Q-factor, and resonance frequency range change as a function of their cross-sectional aspect ratios. In order to conduct resonance measurements of the resonators, we selected a microstrip ring as an antenna. After optimizing the geometrical dimensions of the microstrip ring to provide a maximum electric field in the coupling gap, our model RF metastructure resonator was placed over the gap region to couple it in the near field. Following the completion of the numerical solutions, the systematic approach’s next phase involved implementing the RF metastructure’s fabrication process. In addition to the two-photon lithography technique, thick-film metal deposition with electroplating and seed layer removal with dry etching were included to implement the numerically simulated structures.

**Fig. 1:**
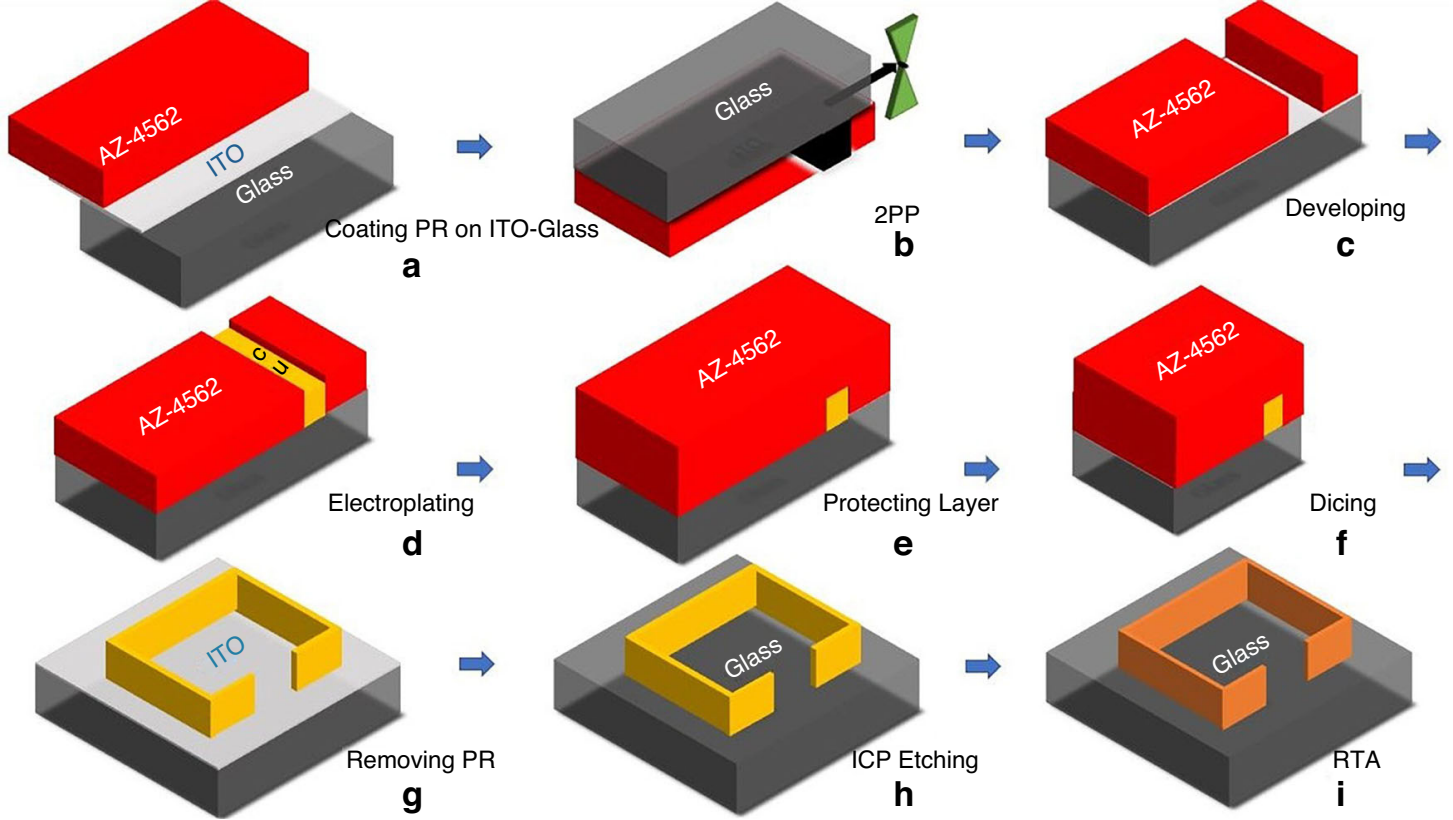
**Proposed process flow**. The developed fabrication methods consist of the following steps: **a** Spin-coated AZ-4562 positive photoresist over ITO-coated glass, **b** placing the prepared substrate on the sample holder of the 3D printing system and exposing light to obtain the desired pattern, **c** developing the exposed part of the photoresist, **d** thick film deposition of copper metal over ITO seed layer along the line of the given pattern, **e** spin-coated the protecting layer, **f** cutting the substrate into smaller pieces with a dicing saw, **g** removing the photoresist, **h** dry etching of the ITO seed layer with ICP, and **i** thermal annealing to strengthen copper structure

We have selected indium tin oxide (ITO) coated glass as the substrate as a need for a transparent conductive seed layer (Fig. 1a). The thickness and the conductivity of ITO over the glass were ~100 nm and 16 Ω per square, respectively. After cleaning the substrate, a positive photoresist (AZ-4562, MicroChem Corp.) was spin-coated onto the substrate (at 2000 rpm for 40 s). Subsequently, the coated substrate was subjected to a pre-baking process (at 110 °C for 150 s).

We established deep trenches using the μFAB-3D system from Microlight3D, based on two-photon polymerization (2PP) direct laser writing. The Microlight3D technology uses a picosecond pulsed laser that emits green light at 532 nm. The writing resolution in the plane is approximately 200 nm, and the vertical resolution is approximately 600 nm. Here, the 2PP technique is utilized as the non-linear light intensity of the simultaneous absorption of two photons while creating the pattern. The chemical reaction, that is functionalization of diazotnaphthoquinone (DNQ) and carboxylic acid groups (COOH), starts with the help of the laser having 532 nm wavelength at the voxel (small volume near the focal spot) of this photosensitive material. The chemically activated exposed region becomes more soluble and is then removed by a developer solution to reach the desired pattern. We can modify the trajectory of the laser beam, which determines the laser’s path for the photosensitive material’s chemical reaction. Modifying the path enables us to move the voxel in any direction during the patterning procedure, thereby granting us 3D-patterning capabilities. Furthermore, this adjustment also facilitates the attainment of flat sidewalls of deep trenches, which is an important requirement for implementing designs with high-aspect ratios.

As shown in Fig. 1b, we placed the prepared sample face-down to avoid diffraction from the substrate and provide steeper deep trenches. During the exposure of the photoresist, the laser focal point was moved from the top to the bottom layer through photosensitive material to increase the design quality. Laser gain (power corresponding to the used objective and photoresist), exposure time, and trajectory are critical to implement the desired 3D-designs. After adjusting all these parameters specifically for our pattern specifications (laser gain: 0.055, exposure time: 1.5 ms, filling interior horizontal/vertical: 1 µm), the printing was completed.

**Fig. 2:**
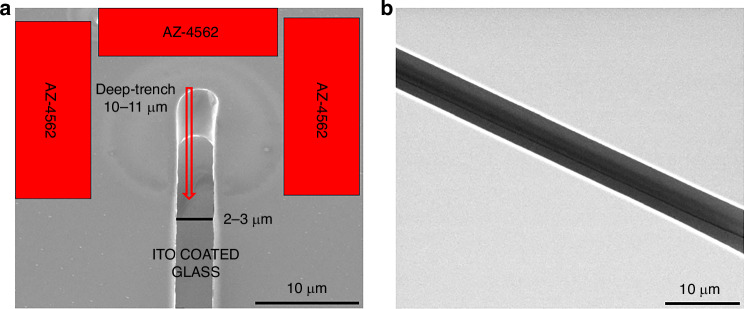
**High aspect ratio design pattern**. **a** 10–11 µm deep third-dimension and 2–3 µm width and **b** flat sidewalls. (Scale bar: 10 µm)

The following step in the process flow was the photoresist development to prepare the deep trenches using AZ-400K developer (MicroChem Corp.) mixed with deionized water in a 3:1 ratio for 5 min (Fig. 1c). This solution rate was deemed necessary due to the high-aspect-ratio structure of the sample, which required a rapid development of the exposed region without the risk of over-development ruining the flatness of trenches. The developed samples had a high-aspect ratio, a width of 2–3 µm, and a thickness of 10–11 µm, as displayed in Fig. 2a, b. At the metallization stage over the deep trenches, the electroplating technique was preferred for thick-film deposition, which provides an advantage in implementing of complex 3D structures (Fig. 1d).

The setup for metal film deposition comprises a voltage source, a copper anode, a hot plate stirrer, wires, and a copper electroplating solution. In the electroplating process, we utilized a Keithley 2400 as the voltage source, which allowed us to administer customized voltage pulses for our electroplating process. Here, the voltage source with current limiting capability was preferred to move copper ions from the copper plate (anode) to the ITO seed layer (cathode) within the high-speed bright copper electroplating solution. This type of source customization yielded superior results in scenarios involving high-aspect-ratio patterns. Higher temperatures in the electroplating bath provide several important advantages. They enhance the metal deposition rate by increasing ion mobility within the solution, decreasing viscosity, and improving solution flow, leading to better surface uniformity. Additionally, the heated bath solution improves adhesion by strengthening the bond between the deposited metal layer and the substrate. Lastly, higher temperatures help reduce internal stresses in the deposited layers, minimizing issues such as peeling or cracking in the plated film. Besides heating, stirring is also crucial in the electroplating process, as it ensures uniform distribution of the electroplating solution, leading to consistent metal deposition and reducing variations in plating thickness. However, the stirrer speed must be carefully adjusted to prevent uneven plating or damage to the sample. Thus, the bath solution was heated at 50 °C with a low-rate stirrer such as 100–200 rpm. For the current-controlled voltage source output of 100 mV with 100 µA current limitation, we found the deposition rate to be 750 nm/min. Samples with ~2, 4, and 8 µm metal thickness, as shown in Fig. 4, were fabricated by adjusting the electroplating duration.

**Fig. 3:**
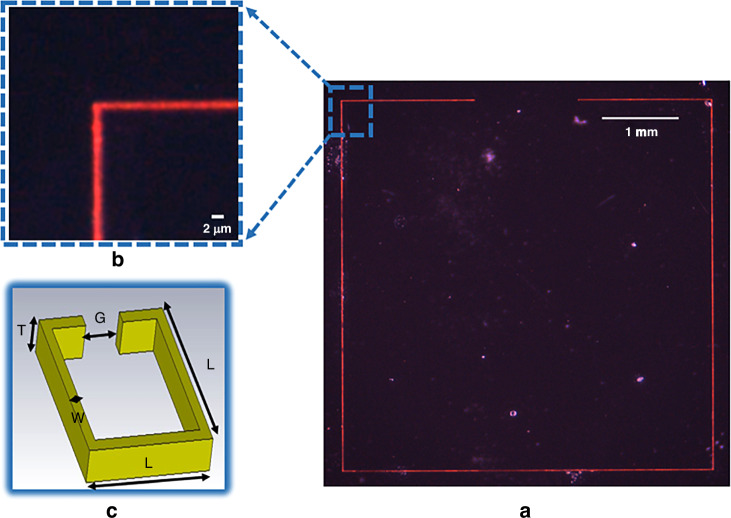
**Our fabricated RF metastructure resonator using the proposed process flow**. **a** Red lines show the metal parts, while dark parts show the glass, **b** zoomed at the corner, and **c** with the physical dimensions of the metastructure which are T:4-6 µm, W:2-3 µm, L:4.3 mm, G:1.2 mm. (Scale bars: 2 µm and 1 mm, respectively)

After thick metal film deposition, we diced the substrate into pieces, each size 8 mm $\times$ 8 mm in size to take experimentally accurate measurements by using a dicing saw. Since the samples had high-aspect-ratio metal structures, AZ-4562 photoresist as a protection layer was coated to protect the metal structures during the dicing operation, shown in Fig. 1e, f. After the removal of the photoresist (Fig. 1g), the seed layer, ITO, had to be etched to have the coupling only with the designed metal structures as shown in Fig. 1h. Inductively coupled plasma (ICP) etch was employed. We used Ar and CF4 gases with flow rates of 18 sccm and 2 sccm, respectively, while RF power was set to 500 W, DC power to 300 W, and pressure to 20 mTorr. The etching rate was approximately 15 nm per minute for ITO seed layer. Subsequently, rapid thermal annealing at 500 °C for 10 min was applied to strengthen the bonds of copper, which enhanced the conductivity of the high-aspect-ratio metastructure (Fig. 1i). These high-aspect-ratio structures have lower thermal and mechanical stability due to their material and structural imperfections. By using other conventional metallization methods, they can achieve better film uniformity, resulting in higher thermal and mechanical stability. However, electroplating for metallization offers more flexibility, particularly for complex high-aspect-ratio structures, which is why we preferred this method. Using electroplating in these structures can introduce grain boundaries and impurities, leading to lower thermal stability. Moreover, electroplating of high-aspect-ratio structures can induce internal stresses, reducing mechanical stability. Nevertheless, thermal annealing can help reduce residual stress and improve grain size and bonding, thereby enhancing both thermal and mechanical stability. The length of one side of the resonator, the longest dimension, and the metal width, the shortest dimension, are ~4300 µm and 2 µm, respectively, indicating an enormous ratio of 2150, as illustrated in Fig. 3a, b, c. The proposed method offers unique design capabilities when compared to structures fabricated through other techniques, such as EBL, optical lithography, and NIL.

**Fig. 4:**
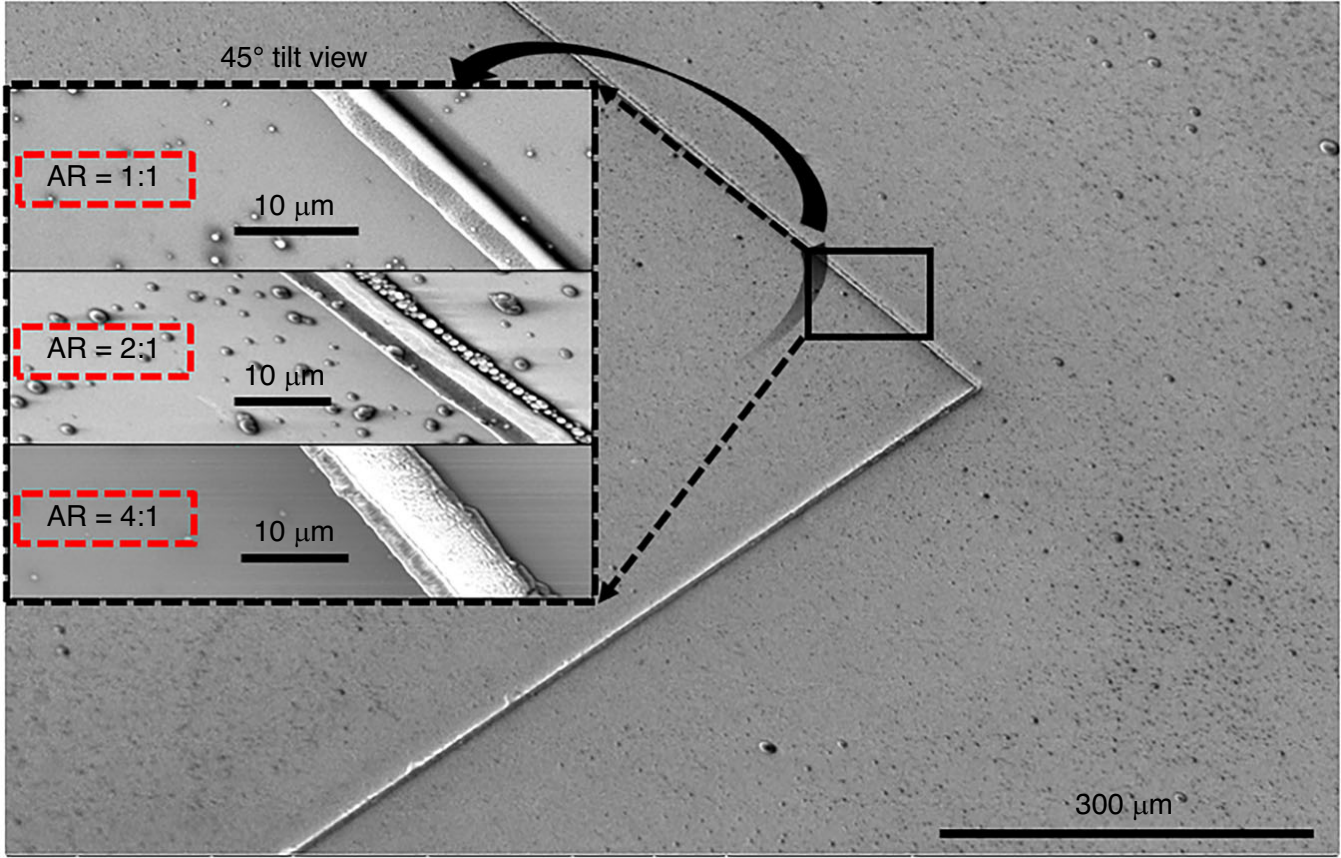
**Scanning electron microscopy images**. Our fabricated RF metastructures, are shown with the aspect ratios of the metal parts, which are 1, 2, and 4, respectively. (Scale bars: 10 µm and 300 µm, respectively)

Deep trenches are shown in Figs. 3 and 4. Here, adjustments to the electroplating process, which are the operational parameters of current-controlled voltage source, heating and stirring the solution, enhanced the uniformity in the deposition process. Heating the solution also increases the electroplating deposition rate, which means heating can lead to thicker coating at a particular time. Thus, the following optimization of time duration has to be considered together with the temperature of the solution. Also, high stirring rates can damage structures with the mixing force of the liquid since high aspect-ratio structures are fragile. The SEM images of our fabricated structures in Fig. 4 show that the resonators possess nicely flat metal sidewalls and high-aspect ratios of around 1, 2, and 4, respectively.

Our fabrication approach offers the opportunity to build RF metastructures that allow tuning resonance frequency, raise the Q-factor, and reduce the footprint. Metastructure resonators in the resonance frequency range of 4–6 GHz were simulated in CST Microwave Studio to show the importance of the aspect ratio with a comparative analysis of their resonance frequency shift, Q-factor, and footprint as a function of the aspect ratios (metal thickness to width ratio). Then, the designed RF metastructures having distinct aspect ratios were fabricated.

**Fig. 5:**
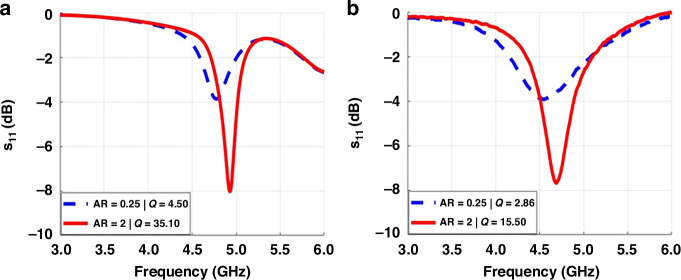
**S**_**11**_ **Spectra**. **a** Numerical simulation results and **b** experimental measurement results. The resonance frequency and Q-factor increase with the increasing metal thickness while keeping metal width fixed

The exact value of the Q-factor can be calculated as the resonance frequency divided by the bandwidth where the reflection coefficient is 3 dB higher than it is at the resonance^35^. Results show that the Q-factor of the resonator with a 2-aspect ratio is 6–7 times larger than the resonator with a 0.25-aspect ratio. Also, we examined a larger resonance frequency shift of 200 MHz varying aspect ratios of the RF metastructure resonators in both numerical simulations and experimental measurements thanks to the third-dimension effect. The resonance frequency rises as the metal thickness of the resonator grows because the product of inductance and capacitance decreases. According to the analytical approach for inductance calculation^32^, as metal thickness grows, the inductive effect diminishes. The capacitive impact, on the other hand, grows according to the capacitance calculation^34^. Because the rate of reduction in the inductor value is larger than the rate of increase in capacitance, the resonance frequency increases with increasing metal thickness. Therefore, using the third-dimension effect from the deep trenches, the resonance frequency of the metastructure designs can be fine-tuned. Here, Fig. 5a, b show frequency tuning and improved Q-factor of the resonators with the aspect ratio.

As a result, we have tested our process flow with the deep-trenched RF metastructures. Implementing RF metastructures also shows the importance of the cross-sectional aspect ratios based on metal thickness in terms of Q-factor and tuning resonance frequency. The result supports the idea of the effectiveness of the deep trenches used in RF metastructures.

The fundamental limits of the aspect ratio that can be achieved using the proposed methods are primarily determined by material properties and the challenges involved in optimizing sufficient energy for a chemical reaction in a focused region from a 2PP perspective. Additionally, there are difficulties in achieving uniform deposition in high-aspect-ratio, large-area structures, which increase instability and fragility as the aspect ratio grows. This can also lead to potential defects in the current flow within the RF structures. These limiting factors affect both the resonance frequency and the quality factor of RF metastructures, constraining their enhancement to a certain extent based on the aspect ratio achieved during the fabrication process. Neglecting these constraints could result in production defects, such as improper metal film deposition or structural damage to the RF elements, compromising their functionality and performance.

**Fig. 6:**
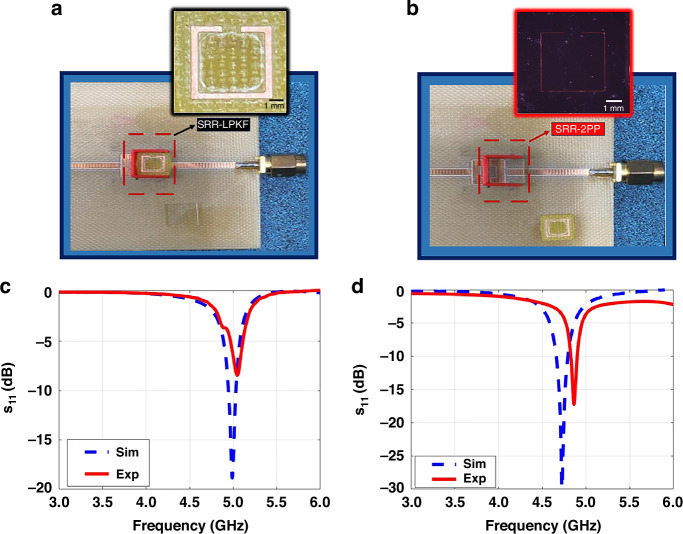
**Two different resonators**. **a** Conventional RF metamaterial resonator fabricated by the PCB milling and prototyping machine, **b** our high-aspect ratio resonator fabricated by our proposed fabrication methods with 2PP; ${S}_{11}$ spectra experimentally measured and numerically calculated for (**c**) the RF metastructure resonator fabricated by the PCB LPKF machine and (**d**) our RF metastructure resonator fabricated by our proposed method. These result show that the length of the resonator decreases from 5.8 mm to 4.3 mm, and the metal width decreases from 0.6 mm to sub-10 µm while increasing the aspect ratio to keep the same resonance frequency range and quality factor

Figure 6 shows two different resonators with the exact substrate sizes: an SRR RF metamaterial, produced using conventional LPKF PCB machine techniques and an RF metastructure created using our proposed methodology. The width of the RF metastructure was determined to be sub-10 µm with a 1.2 mm split dimension and 4.3 mm one-side length. However, the LPKF machine had a constraint on the metal width, with a minimum width of 0.6 mm. Therefore, the resonator, depicted in Fig. 6a, was fabricated with a minimum width dimension of 0.6 mm and a split dimension of 1.2 mm to demonstrate miniaturization. Besides, to adjust the resonator’s resonance frequency, we increased the one-side length to 5.8 mm, as a longer length increases inductance.

The resonance frequency decreases when the metal width of the resonator is reduced from 0.6 mm to sub-10 µm while the aspect ratio remains constant. However, the Q-factor of the resonator was also decreasing in this case. Because of that, we could not obtain the desired resonance frequency. To compensate for this situation, the Q-factor was boosted with the help of high aspect ratio metal structures. Thus, increasing the aspect ratio of the resonator was required to observe resonance frequency for sub-10 µm case by increasing the metal thickness.

Analyzing metal footprints is also essential for circuit implementation in structures with a limited area to obtain a similar resonance frequency with the same range of Q-factor. Figure 6c, d shows that RF metastructure resonator, having the third-dimension effect, acquired the same resonance frequency range of 5 GHz with a better Q-factor when the width of the metal structure was reduced from 0.6 mm to sub-10 µm as depicted in Fig. 6a, b. Here, although the length of one side of the SRR-LPKF is 5.8 mm, one side of the square RF metastructure is 4.3 mm. The findings reveal that RF metastructures using 3D-printing have small device footprints. This means that the resonator’s footprint was reduced from 33.64 to 18.49 $m{m}^{2}$, a reduction of 45%.

## Conclusions

In conclusion, we have demonstrated the proposed fabrication method by combining a 3D printing system based on 2PP with electroplating for thick film metal deposition and dry etching for the seed layers. 2PP provided us with the ability to define high aspect ratio patterns with deep trenches. Electroplating allowed for metal film deposition through the deep trenches, and the dry etching process helped to have only a patterned copper conductive layer over the substrate. To demonstrate the developed process flow, we fabricated deep-trenched RF metastructure resonators concerning the depth effect as the third dimension. The fabricated RF resonators are compared with numerical analysis to show fabrication process flow works well. The effect of the metal thickness was analyzed to observe variations in Q-factor and resonance frequency. Furthermore, the proposed new fabrication process flow is used to miniaturize metal footprint size, increase the quality factor, and tune the resonance frequency in 3D-RF metastructures. The findings of our experiments indicate that the method we proposed, involving the creation of deep trenches through 3D-printing, presents a promising avenue for the fabrication of intricate metal-based structures with high aspect ratios.

## Supplementary information

Supporting Information
