# Supplementary material for: Micro-3D sculptured metastructures with deep trenches for sub-10 μm resolution
Source: Microsyst Nanoeng. 2025 Mar 12;11:47. doi: 10.1038/s41378-025-00888-5 (PMC11897358; doi:10.1038/s41378-025-00888-5)
Supplement: Supplementary file 1 — Supporting Information [file 41378_2025_888_MOESM1_ESM.docx]

**Supporting Information**

**Micro-3D Sculptured Metastructures with Deep Trenches for Sub-10 μm Resolution**

*Anıl Çağrı Atak^1^, Emre Ünal^1^, and Hilmi Volkan Demir^1, 2^**

A.Ç. Atak, E. Ünal, Prof. H. V. Demir

[1] Department of Electrical and Electronics Engineering, Department of Physics, UNAM – National Nanotechnology Research Center and Institute of Materials Science and Nanotechnology, Bilkent University, Ankara 06800, Turkey

*E-mail: [volkan@bilkent.edu.tr](mailto:volkan@bilkent.edu.tr)

Prof. H. V. Demir

[2] Luminous! Center of Excellence for Semiconductor Lighting and Displays, School of Electrical and Electronic Engineering, Division of Physics and Applied Physics, School of Physical and Mathematical Sciences, School of Materials Science and Engineering, Nanyang Technological University, Singapore 639798, Singapore

*E-mail: [hvdemir@ntu.edu.sg](mailto:hvdemir@ntu.edu.sg)

Keywords: three-dimensional printing, deep trenches, two-photon polymerization, RF metastructure

**Metastructure Part**

Our main aim is to develop and demonstrate our fabrication process flow that is based on constructing micro-3D-sculptured deep trenches, electroplating for thick metal film deposition and dry etching to remove the seed layer to implement the design of our RF metastructures. For this reason, we chose our proof-of-concept demonstration model to be high aspect ratio RF metastructures that apply the third-dimension effect to well-known two-dimensional RF metamaterials with sub-10 µm resolution. The idea of RF metastructure comes with the concept of SRRs, well-known RF metamaterials, including one or more metallic rings with a split. These resonators are excellent candidates for transition from planar to 3D designs with the extrusion of vertical dimension. To this end, we selected this class type of RF metastructures as the test model also to demonstrate the importance of high aspect ratio metal partials, generally ignored, in analyzing SRRs. In designing our RF resonators, we numerically simulated metastructures using full electromagnetic solutions (CST Microwave Studio). Their resonance frequencies were intended to be placed in a targeted frequency range while undertaking comparative analyses concerning the resonance frequency shift, Q-factor, and overall footprint size as a function of their cross-sectional aspect ratios. Furthermore, resonance characterization of our resonators is required, and we selected a microstrip ring architecture as an antenna to experimentally perform their measurements.

The resonance frequency of our resonator can be calculated using the following geometrical parameters based on LC equivalent circuit model^1-3^ :


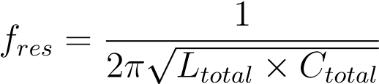
 (1)


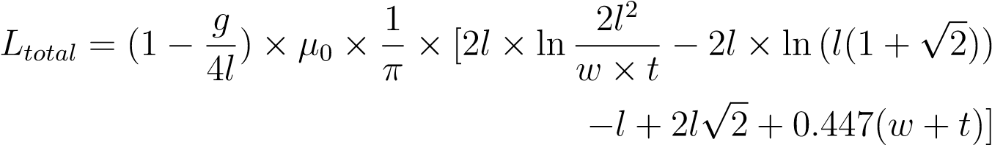
 (2)


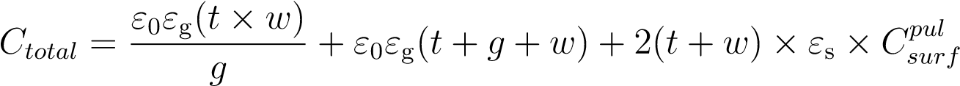
 (3)


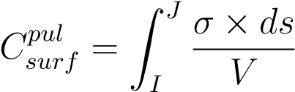
 (4)

where

*ε*_0_: free-space permittivity

*ε*_g_: effective dielectric constant for substrate

*ε*_s_: effective dielectric constant for metal ring

*µ*_0_: free-space permeability

t: metal thickness

w: metal width

g: split-gap of the resonator

l: length

*C_surf_^pul^* : surface capacitance per unit length

*σ*: surface charge density

ds: integral element of length (differential length) of the loop

V: potential difference between opposite points of the loop

As mentioned above, the varied geometric dimensions were set in all parts and applied to analyze and understand the importance of the cross-sectional aspect ratio in the resonance behavior.

The Q-factor can be calculated using the 3 dB difference spectral points around the resonance frequency^4^:


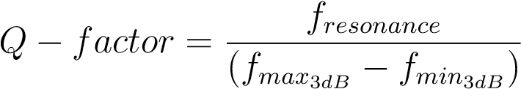


**Microstrip Ring**

A microstrip ring was used for experimental measurement of our fabricated RF metastructure ring resonator with the help of near-field coupling. In designing the microstrip ring, an FR4 substrate with 1.6 mm thickness and 4.3 dielectric constant was used. This consists of a ground plane (backside of the PCB), feed lines, and a planar microstrip ring (frontside of the PCB). Fig 1. (a) and (b) show the microstrip ring and its dimensions. Moreover, the SMA connector was designed for driving to obtain more accurate results between the simulation and experiment.

The location where our fabricated resonator is to be placed over the microstrip ring is essential for the sensitivity and accuracy of the measurement. Thus, electric field analysis was performed in the simulation tool to find where the electric field was more localized. The fabricated resonator should be located where the electric field (E-field) is the strongest.

Fig. 2 shows that the electric field is more concentrated in the coupling gap. The distance of the coupling gap was optimized by decreasing the gap dimension to obtain a strong E-field in this region^5^.

Fig. 3, the microstrip ring’s result and the glass substrate’s effect are observed when we place it over the coupling gap.

**Experimental Setup**

The microstrip ring was employed as a tool helping to take measurements of our fabricated RF metastructure resonators. Thus, these resonators were placed over the coupling gap, and we observed the resonances in S-parameters. Here, the microstrip ring was used as an antenna to detect the resonances experimentally. In Fig. 4, the experimental setup with the resonator is depicted to be numerically simulated.

E-field analysis is shown in Fig. 5 to comprehend how to find resonance frequency with this experimental setup. When we place the resonator over the coupling gap, there is a strong electric field in the gap.

The resonance frequency due to our RF metastructure resonator is found around 5 GHz in the reflection coefficient. Also, the cross-sectional aspect ratio effects on the resonance frequency and Q-factor using this experimental setup are found in the numerical simulations, observable in Fig. 6.

Fig. 6 shows that there is no resonance frequency around 5 GHz when the glass substrate, which does not have a metastructure on it, is placed over the coupling gap of the microstrip ring. However, when the same glass substrate has the metal parts of the RF metastructure, it is clearly observed that the resonance exists at almost the same frequency as in the cases of using the simulation setup 1 and 2. In addition, the behavior of the resonance because of the third dimension effect is similar to our previous numerical results, which show increased resonance frequencies with the increasing aspect ratios while having the split gap (1.2 mm) and metal width (3 µm) fixed.

Following the numerical analyses regarding the effect of dimension variation of RF metastructure, we can move onto our main aim, which is the development of the proposed fabrication technique along with the recipes of every fabrication step and the entire process flow to construct micro-3D-sculptured deep trenches, electroplating for thick metal deposition and dry etching for the seed layer removal.

**Fabrication Part**

At the phase stage of the fabrication process for the simulated RF metastructures by merging the 2PP to construct micro-3D-sculptured deep trenches and other micro-fabrication methods as fundamentally electroplating and dry etching.

**Coating Photoresist on ITO-Glass**

We have considered that there are some requirements in the selection of our substrate. The starting has to be conductive and transparent due to the need for a seed layer in the electroplating process and the effective use in the 2PP process. Thus, we selected indium tin oxide (ITO) coated glass as the substrate. The thickness and the conductivity of the ITO film over the glass were approximately 100 nm and 16 Ω per square, respectively. After cleaning the substrate, we spin-coated positive photoresist AZ-4562 (MicroChem Corp.) on the substrate at 2,000 rpm for 40 s. Then the pre-baking at 110° C oven for 150 s was carried out, and the prepared sample was ready to put on the sample holder of the Microlight3D.

**Two-Photon Polymerization (2PP)**

Based on 3D printing technology, as the main fabrication step of our process flow, we use a 3D printing system based on two-photon polymerization to build our high aspect ratio pattern. Here, we established deep trenches using this two-photon lithography 3D printing system.

The 2PP technique is based on the simultaneous nonlinear absorption of two photons to define desired patterns. The system and working mechanism illustrated in Fig. 7 reveal that the required energy for the chemical activation of the photoresist within the focal cone (except for the focal point) cannot be reached. The required energy, which is proportional to the threshold intensity to start the chemical reaction of photosensitive material, is achieved only in the voxel (small volume near the focal spot) part. Here, the chemical reaction, which is the functionalization of diazotnaphthoquinone (DNQ) and carboxylic acid groups (COOH), starts with the help of the laser at 532 nm wavelength at the focused voxel of this photosensitive material. The chemically activated exposed region becomes more soluble (because it a positive photoresist) and is removed by a developer solution to leave a desired pattern. We can modify the trajectory of the laser beam, which determines the path along which the photosensitive material’s chemical reaction to take place. Modifying the path enables us to move the voxel in any direction during the patterning procedure, thereby equipping us with a 3D patterning capability. Furthermore, this adjustment also facilitates the attainment of flat sidewalls of deep trenches, which is an essential requirement for implementing designs with high aspect ratios.

To proceed to the 3D fabrication part, we need to compute trajectories by slicing operation. The UPLSAPO 20x objective having 0.75 numerical aperture is chosen among 10x, 20x, and 100x objectives for our design specifications. Here, the main part arranges lateral and vertical sizes through the utilized objective and pattern specifications.

The slicing direction was chosen [0 0 1] because the structures have a deep third-dimension effect in the z-direction, and this direction is more appropriate for our case. The slicing process was applied to define the paths through the structure’s walls. Here, an adaptive layer thickness was also selected. It is related to the diminishing of the staircase effect. In our case, there was no possible staircase effect, but a structure having ovality needs this adjustment to save itself from the staircase effect. Thus, the use of adaptive layer thickness is generally preferable. In addition, merging trajectories are generally used to reduce the number of paths, positively contributing to the fabrication speed. The hatching option was also turned on. This is responsible for nearly planar 3D shapes having less than a 5° critical angle and gives extra pathing for both the bottom and top sides of the structure. It decreases the chance of non-exposed parts of the structure at the interfaces, contributing to quickly developing the structure’s procedure. The curing of walls with those slicing options is sufficient to obtain a pattern since the curing of those provides hardness, and the liquid part remains trapped interior of solid parts. This provides a faster fabrication procedure because of having fewer paths.

On the other hand, in our case, we used AZ-4562 positive photoresist. Therefore, we needed paths to expose the interior part of the structure. Optimizing the filling of interior parts was essential to obtain an entire structure. If the laser trajectory uses too intense beam in general, there might be some voxel parts that burn the photoresist, which later the developer does not remove. In order to prevent the photoresist burning, laser trajectory should be well-defined and optimized according to the structure, the photoresist type and the thickness.

In summary, 1-1.25 µm voxel sizes and 50-60% voxel overlap with slicing having adaptive layer thickness, hatching and filling interior having 1-1.25 µm on both distance lines and distance layers were performed. After finishing the computational part of the trajectory, it should be exported for another software package Lithos. Here, the objective’s numerical aperture and the resin’s refractive index had to be entered as 0.75 and 1.56, respectively. While the laser gain was constant, path sorting should start from the top since we placed the prepared sample face-down to avoid diffraction from the substrate and generated steeper and deep trenches.

Focal point adjustment is crucial to determining the interfaces and designing the procedure through Lithos software. Locating the focal point at the correct interface, which is the interface between the substrate and the photoresist, was required. Otherwise, we could not obtain the pattern properly. During this adjustment procedure, we selected an opaque substrate and correct objective magnification as the fabrication parameters. Although a transparent substrate was utilized in our process flow, the reason for choosing an opaque substrate in the system is due to the flip-down configuration of the sample like an opaque substrate to prevent diffractions from the substrate. We used the camera systems of the Microlight3D to find the location of the focal point by changing the z-axis. After adjusting the focal point, the dosage matrix was applied by changing laser gain from 0.200 to 0.020 with the exposure range of 500 µs – 10000 µs. From the dosage matrix, the appropriate laser gain and exposure range were determined to be 0.055 laser gain and 1500 µs exposure time, respectively. We applied them as the design parameters in our implementation.

**Developing Procedure**

The next step in the process was to develop the photoresist to leave the deep trenches using AZ-400K developer (manufactured by MicroChem Corp.) mixed with deionized water in a 3:1 ratio for 5 min. This solution rate was determined by the rapid development of the exposed region because our structure having a high aspect ratio pattern, and it needs rapid development without ruining the flatness of trenches due to over-developing. At the end of this step, the pattern of the RF metastructure was defined with the help of the 3D printer, ready for the following fabrication steps.

**Electroplating Procedure**

Electroplating plates a metal on a structure. In this process, metal ions in the electroplating solution are transferred from an anode to a cathode, which is coupled to a lower electric potential. The electroplating setup consists of a power source, a copper anode, a copper cathode sample having the seed layer, wires, a hot plate stirrer, and a copper electroplating solution.

In the plating procedure, customized current and voltage need to be applied by a source (Keithley-2400). The copper anode is connected to the positive terminal of the source, and the cathode touching the seed layer of the substrate is connected to the negative terminal of the source. They are inside the copper bath solution (high-speed bright copper electroplating solution manufactured by Sigma-Aldrich). This copper electroplating solution consists of cupric sulfate and sulfuric acid with a small amount of hydrochloric acid and organic additives. It is appropriate for patterns having trenches and vias.

The prepared device structures, which had already been developed and had a RF metastructure pattern, were ready for the electroplating procedure. There was a need for a conductive surface on the device to conduct a current. Thus, a small portion of the photoresist was removed with acetone from the face of the device. Also, it is worth emphasizing that the cleanliness of the seed layer is critical for depositing metal parts. Before starting electroplating, cleaning with DI water and descumming are generally advised. After cleaning, the cathode connection was supplied with the ITO seed layer.

The location of the cathode and anode in a beaker is vital for the deposition quality. They must be located vertically and facing each other. It is especially vital for high aspect ratio structures and trenches. Otherwise, some parts of the pattern can have thicker metal parts, and some can have thinner ones, leading to non-uniformity and broken portions. In addition to providing a proper location, we used a hot plate to heat the electroplating solution at 50°C because heating the bath solution provides the required energy for ions/atoms to move around in plating with a stirrer mixing of the copper ions. Using a high stirrer rate during the plating duration results in a non-uniform metal deposition. For this reason, a high stirrer rate was used only before turning on the source, and then it was reduced to 100 rpm. Our electroplating setup is displayed in Fig. 8.

In summary, controlling the height of the plated metal thickness is essential in the electroplating process when using a current source. However, there was a problem related to the uniformity of the plated metal. Using a stirrer mixes copper ions around, and the deposited metal’s height was similar in all parts of the structure, but this caused highly non-uniformed electrodeposition. In the light of the know-how, we move to use the voltage source. The controlled voltage provides very uniform deposition. In addition, adding current limitations and using a stirrer at a very low rate along with the voltage source allow for controlling the uniformity of the deposited metal thickness.

The recipe of using the voltage source, 100 – 150 mV voltage was applied while having a current limitation of 100 - 105 µA and a heated solution bath at 50°C. Also, we needed to be sure about the cleanliness of the seed layer and vertically placed anode/cathode parts. The stirrer was used before turning on the source with a high rate to mix ions well. Then, it was stopped, or it was decreased to 100 rpm. This recipe had a 750 - 1000 nm-per-min deposition rate in our case.

**Protecting Layer Procedure**

Since samples had high-aspect-ratio metal parts, AZ-4562 photoresist as a protection layer was coated to protect the resonator’s metal parts from any damages during the dicing operation. We spin-coated positive photoresist AZ-4562 (MicroChem Corp.) on the substrate at 2000 rpm for 40 seconds. Then the pre-baking at 110° C oven for 150 seconds was carried out.

**Dicing Procedure**

After thick metal film deposition, we cut the substrate size 8 mm x 8 mm to take experimentally accurate measurements through numerical solutions by using a dicing saw. In the cutting procedure, the DAD 3220 machine was used. In here, the work shape is adjusted as square with the 1.1 mm work thickness including 0.11 mm tape thickness. Also, the depth steps for the blade 0.250 with the slow speed.

**Removing Photoresist Procedure**

After completing dicing operation, the photoresist had to be removed from our sample to get ready for seed layer etching purpose. It has removed with the help of the acetone in 1 – 2 minutes. Then, it was put inside of a beaker containing isopropanol softly.

**Inductively Coupled Plasma Etching**

Inductively coupled plasma (ICP) reactive ion etching system was chosen as the tool for our dry etching methods. This system consists of an RF coil with a 13.56 MHz power supply and two RF platen powers connected to the coil and the electrode. Different available gases such as *SF*_6_, *C*_4_*F*_8_, *CHF*_3_, *CF*_4_, *Ar*, and *O*_2_ can flow in plasma generated by the RF generator. The system is characterized by low operating pressure; so, it has one mechanical pump and one turbo pump to bring pressure down to the 10^−6^ Torr level.

In the etching mechanism, we used 20 sccm total gas flow rate, consisting of 18 sccm Ar and 2 sccm *CF*_4_, while the substrate temperature was adjusted to 40°C and the system pressure was 20 mTor. Here, applying RF power increases the etch rate by dissociating more actively *CF*_4_ and increasing the plasma density, and DC-bias accelerates ion bombardment and make more ions reach the ITO surface^6^. According to our ICP system’s specifications, approximately 100 nm ITO layer was removed with 500 W RF power and 300 V DC-bias voltage metastructures.

**Rapid Thermal Annealing**

After being sure of the etching of the ITO surface, we applied rapid thermal annealing to the sample at 500°C for 10 min, which helped strengthen the bonds of copper and enhanced the conductivity of our high aspect ratio

**Scanning electron microscopy**

SEM images of the fabricated metastructures were taken with FEI Quanta 200 FEG ESEM. Those structures were displayed from the top with operational adjustments at 5 – 10 kV and 3.0 – 4.0 spot sizes.


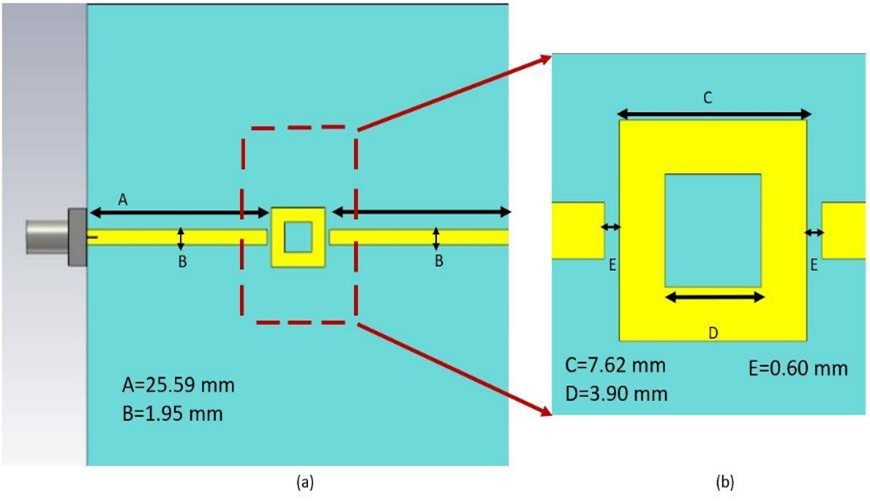


**Fig. 1.** Microstrip ring: (a) microstrip ring with the feed line and (b) its zooming along with the dimensions for the microstrip ring geometrical parameters.


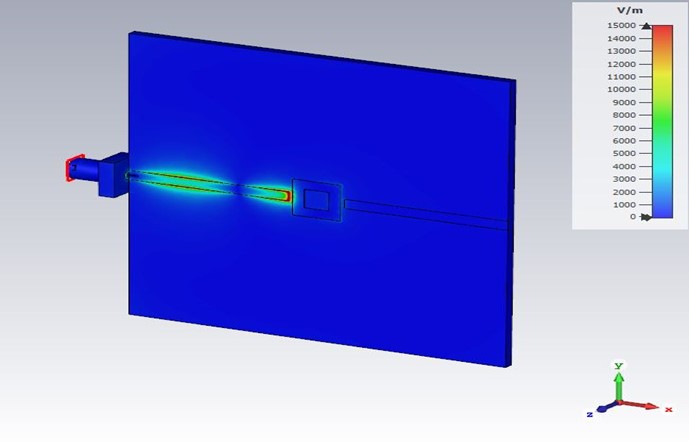


**Fig. 2.** Electric field map of the microstrip ring.


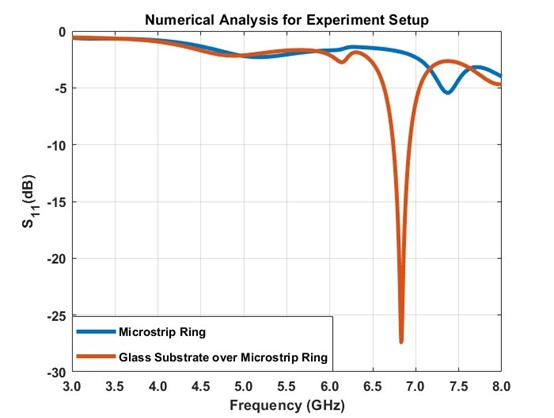


**Fig. 3.** Numerical analysis for the microstrip ring and the glass over the microstrip ring.


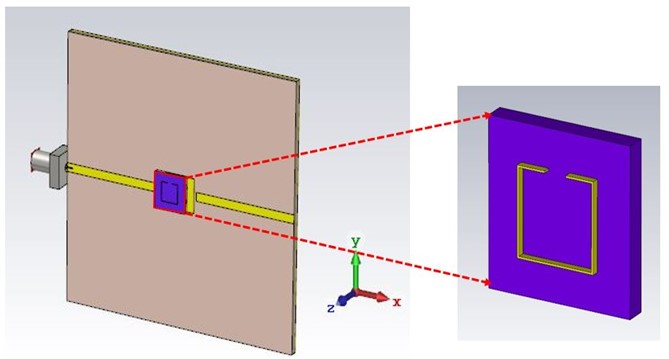


**Fig. 4.** Numerical simulation of the experimental setup with our RF metastructure resonator on it.


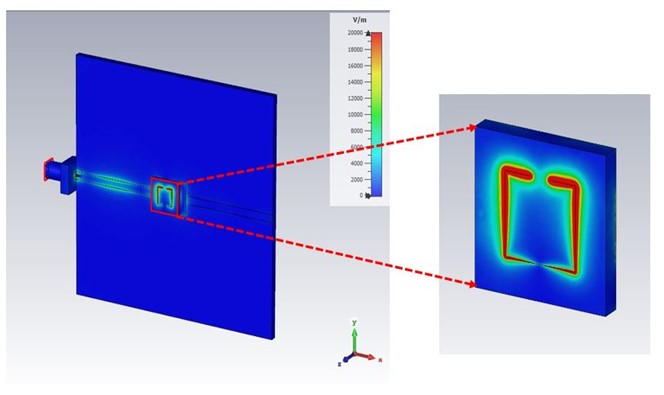


**Fig. 5.** Electric field distribution of the experimental setup with our RF metastructure resonator on it.


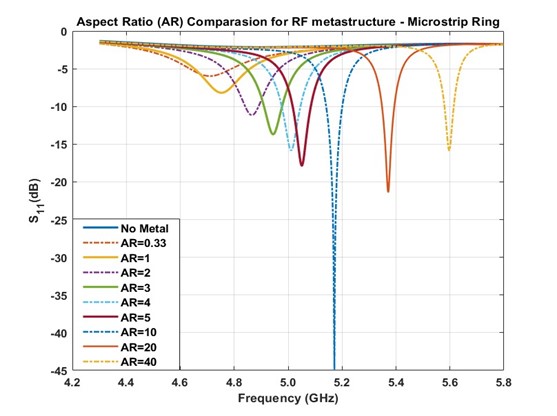


**Fig. 6.** Numerical simulation for the aspect ratio effect for our RF metastructure using the microstrip ring.


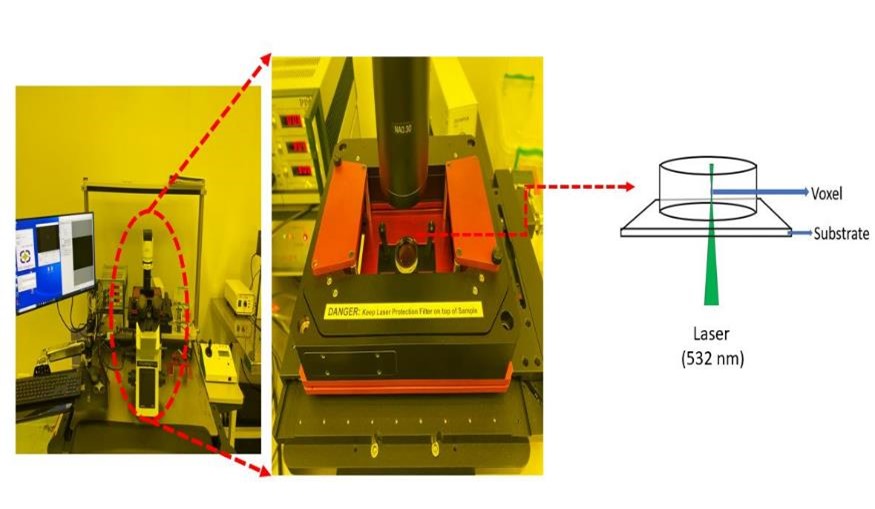


**Fig 7.** The Microlight3D Printer based on 2PP and its general mechanism.


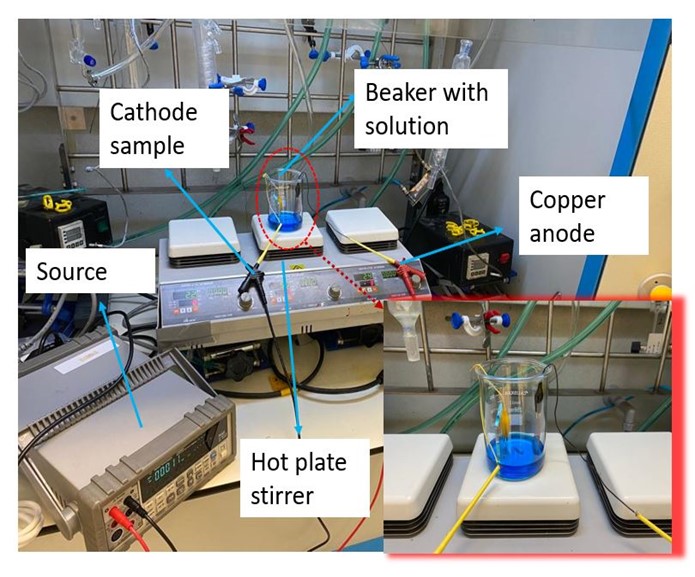


**Fig 8.** Our electroplating setup.

**References**

1. A. Vallecchi, E. Shamonina and C. J. Stevens, *Journal of Applied Physics*, 2019, **125**, 014901.
2. O. Sydoruk, E. Tatartschuk, E. Shamonina and L. Solymar, *Journal of Applied Physics*, 2009, **105**, 014903.
3. S. K. Samanta, R. Pradhan and D. Syam, *Journal of the Optical Society of America B*, 2021, **38**, 2887.
4. F. Iza and J. A. Hopwood, *IEEE Transactions on Plasma Science*, 2003, **31**, 782–787.
5. A. Bogner, C. Steiner, S. Walter, J. Kita, G. Hagen and R. Moos, *Sensors*, 2017, **17**, 2422.
6. H.-S. Kim, J.-C. Woo, Y.-H. Joo and C.-I. Kim, *Vacuum*, 2013, **93**, 7–12.
